# Ileocecal valve-sparing surgery for duplication cysts in the terminal ileum: two case reports and literature review

**DOI:** 10.1186/s40792-022-01483-w

**Published:** 2022-07-06

**Authors:** Koichi Deguchi, Ryuta Saka, Miho Watanabe, Kazunori Masahata, Motonari Nomura, Masafumi Kamiyama, Takehisa Ueno, Yuko Tazuke, Hiroomi Okuyama

**Affiliations:** Department of Pediatric Surgery, Graduate School of Medicine, Osaka University, 2-2, Yamadaoka, Suita-shi, Osaka, Japan

**Keywords:** Enteric duplication, Ileocecal resection, Ileocecal valve/junction, Cyst mucosectomy

## Abstract

**Background:**

Duplication cysts close to the ileocecal valve are usually treated with ileocecal resection. However, loss of the ileocecal valve will lead to problems, especially in infants. Mucosectomy of the cyst would be a better alternative that preserves the ileocecal valve. We report two cases of duplication cyst in the terminal ileum successfully treated with mucosectomy.

**Case presentation:**

Case 1. A 3-month-old boy with bilious emesis and abdominal distention was referred to our hospital with a diagnosis of small bowel obstruction caused by an abdominal cyst. Computed tomography revealed a cystic mass compressing the terminal ileum and causing mechanical small bowel obstruction. His general condition deteriorated quickly; emergency laparotomy was performed. Although the small intestines were dilated and partially twisted, there was no necrosis. Following intestinal decompression, a cystic mass adjacent to the terminal ileum was confirmed on the mesenteric side. Cyst mucosectomy was performed to preserve the ileocecal valve. Case 2. A 5-month-old boy with sudden onset of hematochezia was referred to our hospital with a diagnosis of intussusception. Following unsuccessful contrast enemas, emergency surgery was performed. A cystic mass adjacent to the terminal ileum was confirmed; there was no intussusception. Cyst mucosectomy was performed. Both patients had an uneventful postoperative course.

**Conclusions:**

Cyst mucosectomy, which preserves the ileocecal valve, is safe and effective for treating duplication cysts in the terminal ileum.

## Background

Duplication cyst of the alimentary tract is a rare congenital anomaly with diverse clinical presentation [1, 2]. Surgical resection of the cyst is warranted due to potential complications, such as small bowel obstruction, bowel perforation, ectopic gastric mucosa, and risk of malignancy [3]. There are two major surgical approaches for duplication cysts: (1) total cyst excision with resection of adjacent bowel and (2) IC valve-sparing surgery: complete cyst enucleation, which is the full-thickness resection of the duplication cyst, or mucosectomy, which is the removal of the cystic mucosa layer alone, without resection of the native bowel. Because most duplication cysts in the small intestines lie on the mesenteric side of the bowel and share a muscular wall and vasculature with the native intestines, the former approach is preferred for duplication cysts in the small intestines [4–6].

To date, there have been few case reports of IC valve-sparing surgery to preserve IC valve function. Most cases have been treated with IC resection and ileocolic anastomosis, even when IC valve-sparing surgery is feasible [7, 8]. Herein, we report two cases of duplication cyst in the terminal ileum adjacent to the IC valve that were successfully treated with cyst mucosectomy.

## Case presentation

### Case 1

**Fig. 1:**
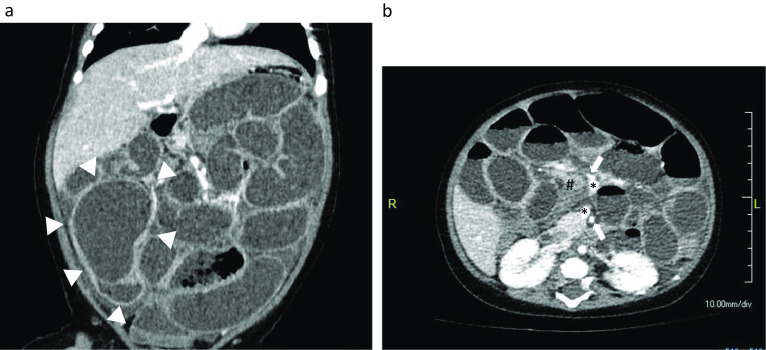
Contrast-enhanced computed tomography images of Case 1. **a** Cystic mass (50 mm, arrowheads) in the terminal ileum with compressed normal ileum was observed. **b** Stretched edematous mesentery (#) caused by twisted and dilated small intestines was observed (arrows: superior mesenteric artery; *superior mesenteric vein)

A previously healthy 3-month-old boy with sudden onset of bilious emesis and abdominal distention for 1 day was transferred to our hospital. Abdominal ultrasound and contrast-enhanced computed tomography showed a cystic mass (60 mm) located in the right lower abdomen (Fig. 1) and mechanical small bowel obstruction. Emergency laparotomy was indicated for small bowel obstruction caused by the IC duplication cyst.

**Fig. 2:**
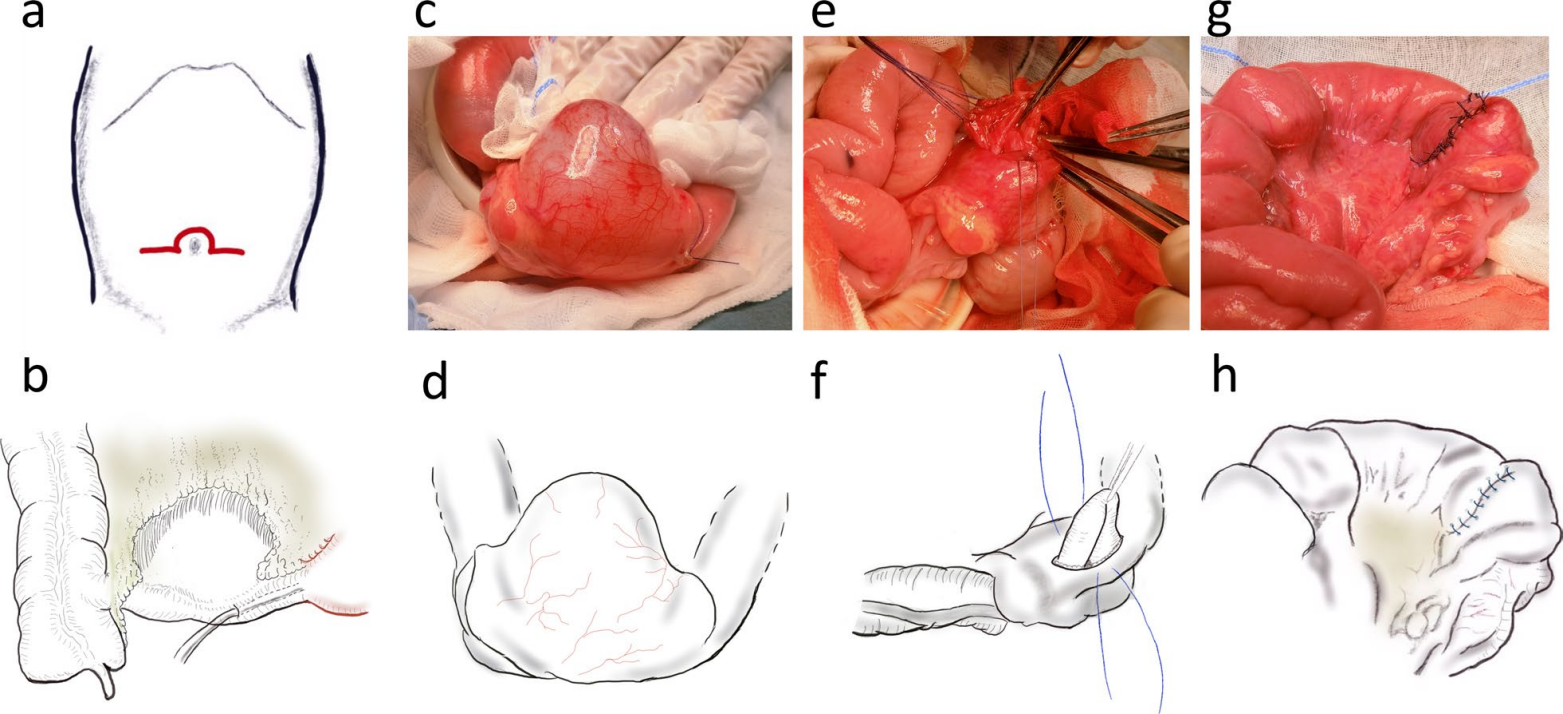
Operative findings and illustration of cyst resection in Case 1. **a** Omega-shaped incision of the umbilicus (length: 9 mm) was made. **b** Schematic showing the location of the duplication cyst. **c**, **d** Cyst was on the mesenteric side at the ileocecal junction. **e**, **f** Mucosa of the cyst was dissected from the muscular layer. **g**, **h** Seromuscular defect was closed in a transverse fashion and the mesentery was repaired

Surgery was performed through an omega-shaped incision of the umbilicus (9 mm in length, Fig. 2a). There was a fair amount of serous ascites. Intestinal torsion due to the duplication cyst was observed; it was corrected manually. Because there was no ischemic bowel, no bowel resection was required. The cyst was located on the mesenteric side of the terminal ileum. The cyst was approximately 50 mm in diameter (Fig. 2b). We attempted to preserve the IC valve even though the cyst was adjacent to the IC valve. We performed mucosectomy of the cyst instead of enucleation to preserve the cyst wall that was shared with the native intestines and to avoid postoperative stricture. Following incision of the mesenteric layer above the cyst, the cyst wall was visualized. We opened the cyst on the mesenteric site. We perform mucosectomy circumferentially from the muscular layer without any injuries to the native intestines with the aid of stay sutures placed in the mucosa (Fig. 2c–f). No communications or injuries between the cyst lumen and the native intestines were confirmed by air leak test. The remaining seromuscular layer was closed using interrupted 4–0 Vicryl sutures (Fig. 2g–h). The operative time was 217 min.

### Case 2

A previously healthy 5-month-old boy with hematochezia was transferred to a local hospital. He was diagnosed with intussusception, but initial multiple enema reductions were unsuccessful. The patient was transferred to our hospital. Another enema reduction was also unsuccessful. Emergency laparoscopic surgery was indicated because of unsuccessful nonsurgical reduction.

**Fig. 3:**
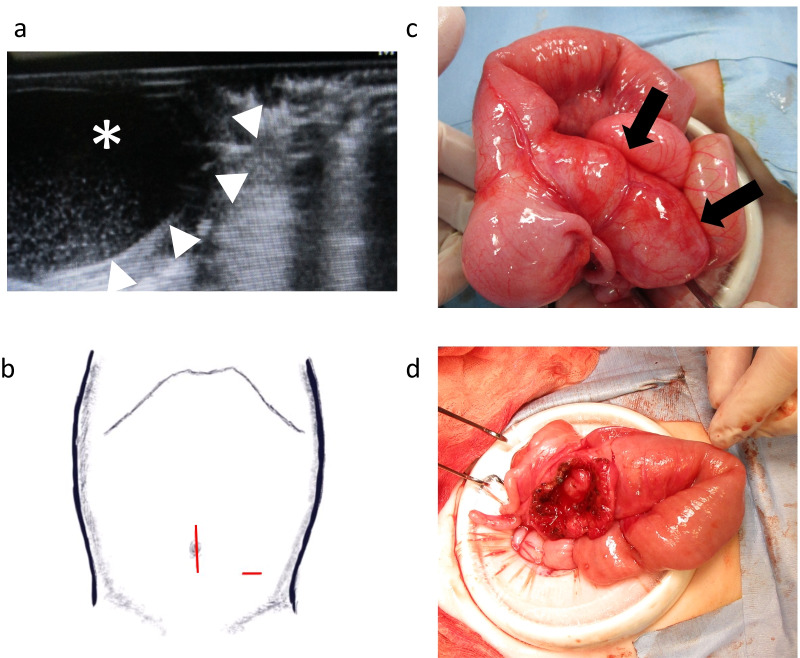
Operative findings in Case 2. **a** Intraoperative ultrasound of the duplication cyst, containing fluid (* and arrowheads). **b** Laparoscopic surgery was commenced with a midline incision at the umbilicus (25 mm in length). An additional 5 mm port was inserted in the left lower abdomen. The midline incision at the umbilicus was then extended to exteriorize the cyst. **c** Cystic mass in the terminal ileum (40 mm, arrows) was observed. We converted to open laparotomy when we detected and diagnosed the mass as a duplication cyst. **d** Mucosa of the cyst was dissected

Laparoscopic surgery was commenced with a midline incision at the umbilicus (25 mm in length, extended later to 30 mm to exteriorize the cyst, Fig. 3b) with a minilaparotomy wound edge protector (Lap-Protector™). No intussusception of the small intestines was observed, but a mass lesion (approximately 40 × 20 mm) was detected on the mesenteric side of the terminal ileum. We converted to open laparotomy. The mass was exteriorized through the incision. Intraoperative ultrasonography confirmed a cystic mass containing serous fluid adjacent to the IC valve that was compressing the native intestines. He was diagnosed with IC duplication cyst (Fig. 3a). The cyst wall was opened with a longitudinal incision. The luminal surface of the cyst was visualized. No communication between the cyst lumen and the native intestines was identified. Mucosectomy was accomplished using electrocautery. One mucosal injury to the native intestines was repaired with a two-layered suture. The inverted duplication cyst was extracted and mucosectomy was completed. After resection of the redundant cyst wall, the seromuscular layer was reconstructed using the remaining cyst wall, as in Case 1 (Fig. 3c, d). The operative time was 204 min.

Both patients had an uneventful postoperative course. Both started oral feeding by postoperative day 5 and reached full oral feeding by postoperative day 8. They were discharged on postoperative day 9 and 8, respectively. There were no adverse events during outpatient follow-up.

## Discussion

The presented cases highlight two major clinical points. First, we performed IC valve-sparing surgery for duplication cysts located in the IC region. Second, we encountered a rare case of IC duplication cyst that manifested as volvulus (Case 1).

The optimal surgical approach for small intestinal duplication cysts is removal of the lesion. This is generally accomplished by resecting the duplication cyst with adjacent bowel and primary anastomosis [9, 10]. In the rare case of a very long tubular intestinal duplication, mucosal resection or marsupialization between the duplication and the native intestines is considered [11]. The optimal surgical procedure for duplication cysts located in the IC region is not well discussed in the literature, despite the importance of a functional IC valve [7]. There are six patients, including ours, who underwent IC valve-sparing resection of IC duplication cysts described in the literature; Table 1 summarizes the clinical and operative data from these cases [7, 8]. All patients had duplication cysts in the IC angle on the mesenteric border. Cyst removal or cyst mucosectomy was successfully performed with uneventful postoperative courses (Table 1). Removal of the IC valve is known to be associated with the following issues: bacterial overgrowth, reduced transit time within the small intestines, impaired absorption, chronic diarrhea, undernutrition, and electrolyte abnormalities [12–15]. Therefore, in the case of IC duplication cyst, we should always consider the possibility of IC valve preservation, especially in neonates and infants.

**Table 1:** Clinical and operative characteristics of patients with ileocecal duplication cyst who underwent ileocecal valve-sparing surgery

Patient	First author	Sex	Presentation	Preoperative diagnosis	Age at surgery	Location of the duplication cyst	Cyst size (mm)	Type of surgery	Site of incision	Operation time (min)	Postoperative complications
1	Catalano [7]	Female	Recurrent vomiting	Intra-abdominal cystic mass	16 days	Ileocecal angle in the mesenteric border	30–50 (Patients 1–3)	Enucleation	Cecal enterotomy on the mesenteric side	Mean, 105 Range 100–110 (Patients 1–3)	None
2	Catalano [7]	Male	Intermittent constipation	Mesenteric versus duplication cyst	14 days	Ileocecal angle in the mesenteric border		Enucleation	Mesenteric side		None
3	Catalano [7]	Male	Vomiting, abdominal distension	Duplication cyst	3 days	Ileocecal angle in the mesenteric border		Enucleation	Mesenteric side		None
4	Endo [8]	Male	Abdominal pain, hematochezia	Duplication cyst	4 years	Ileocecal angle in the mesenteric border	30	Enucleation	Cecal enterotomy on the anti-mesenteric side	146	None
5	Deguchi [current study]	Male	Vomiting, abdominal distension	Duplication cyst	3 months	Ileocecal angle in the mesenteric border	60	Mucosectomy	Mesenteric side	204	None
6	Deguchi [current study]	Male	Hematochezia	Intussusception	5 months	Ileocecal angle in the mesenteric border	40	Mucosectomy	Mesenteric side	217	None

Catalano et al. first reported the safety and efficacy of IC valve-sparing resection in three pediatric patients with IC duplication cyst [7]. They approached the cyst from the mesenteric site. Upon enucleation, they incised the common wall with the native intestines up to the lumen, resected all the common wall, and closed the defect to reconstruct the native intestines. Endo et al. reported another enucleation approach [8]. Since the cyst was located on the mesenteric side and protruded into the lumen of the native intestines, they opened the anti-mesenteric wall of the cecum and visualized the cyst as a submucosal tumor. They successfully enucleated the cyst while preserving the common wall [8]. We incised a layer of the mesentery above the cyst to visualize the cyst wall. We opened the free wall of the cyst on the mesenteric site to reach the lumen of the cyst and dissect the mucosa from the muscular layer. Next, we performed mucosectomy instead of cyst enucleation as described by Catalano et al., because we believe mucosectomy has the advantage of preserving the integrity of the native intestinal wall when the cyst and the native intestines share a substantial muscular layer. We performed mucosectomy successfully and preserved the IC valve with the native intestines intact. Although most duplication cysts do not communicate with the adjacent native intestines [5], meticulous examination of the duplication cyst’s lumen is preferable to rule out any communications or iatrogenic injuries with the native intestines, which we did during mucosectomy. Small communications or injuries could be closed by sutures to avoid leakage from the native intestines.

Concerning clinical presentation, Patient 1 had volvulus and Patient 2 was initially misdiagnosed with intussusception. A review of the Japanese literature on IC duplication showed that the majority of IC duplications were cystic. One-half of the cases manifested by 1 year of age. Initial symptoms included vomiting and abdominal pain. The unique clinical picture of IC duplication cysts included intussusception (34%), hematochezia (24%), and perforation (4%), but no intestinal volvulus has been documented [16]. In Patient 1, the IC duplication cyst compressed the ileum and cecum, causing complete obstruction and eventually acute abdomen. We assume that the dilated ileum and enlarged cyst, together with the loose attachment to the ileum, contributed to elongation of the intestinal loop and partial torsion around the mesenteric axis [17–19]. In Patient 2, although the initial presentation of hematochezia led to a misdiagnosis of intussusception, there might have been ischemic injury of the native mucosa due to compression by the duplication cyst. Pediatricians and surgeons must always be aware of the possibility of a duplication cyst as the cause of hematochezia, intussusception, or intestinal obstruction.

Of note, the choice of surgical approach should be based on the patient’s hemodynamic status, because mucosectomy will require more time than enucleation. The operative times of our cases, which underwent mucosectomy, were 204 and 217 min, respectively. In contrast, operative times in the previous two reports, which underwent enucleation, were 105 min (mean from 3 cases) and 146 min, respectively [7, 8]. Enucleation is suitable for unstable patients to minimize operative time.

## Conclusions

We encountered two patients with IC duplication cyst that presented with volvulus and hematochezia, respectively. IC valve-sparing surgery was safe and effective for duplication cysts adjacent to the IC valve.

## Data Availability

Data for in this report will not be shared, because they include patient information.

## Abbreviation

IC: Ileocecal
